# Decreased Event-Related Desynchronization of Mental Rotation Tasks in Young Tibetan Immigrants

**DOI:** 10.3389/fnhum.2021.664039

**Published:** 2021-06-30

**Authors:** Zu-qiang Xiang, Yi-lin Huang, Guang-li Luo, Hai-lin Ma, De-long Zhang

**Affiliations:** ^1^Department of Psychology, School of Education, Guangzhou University, Guangzhou, China; ^2^The Fourth Primary School of Qiaotou Town, Dongguan, China; ^3^Plateau Brain Science Research Center, Tibet University, Lhasa, China; ^4^Plateau Brain Science Research Center, South China Normal University, Guangzhou, China; ^5^Key Laboratory of Brain, Cognition and Education Sciences, Ministry of Education, Guangzhou, China; ^6^School of Psychology, Center for Studies of Psychological Application, and Guangdong Key Laboratory of Mental Health and Cognitive Science, South China Normal University, Guangzhou, China

**Keywords:** high-altitude exposure, mental rotation, event-related desynchronization, time-frequency analysis, motor cortical excitability

## Abstract

The present study aimed to explore the cortical activity underlying mental rotation in high-altitude immigrants via the event-related desynchronization (ERD), the electroencephalogram time–frequency analysis, and source localization based on electroencephalographic data. When compared with the low-altitude individuals, the reaction time of mental rotation tasks was significantly slower in immigrants who had lived in high-altitude areas for 3 years. The time–frequency analysis showed that the alpha ERD and the beta ERD within the time window (400–700 ms) were decreased during the mental rotation tasks in these immigrants. The decreased ERD was observed at the parietal–occipital regions within the alpha band and at the central–parietal regions within the beta band. The decreased ERD might embody the sensorimotor-related cortical activity from hypoxia, which might be involved in cognitive control function in high-altitude immigrants, which provided insights into the neural mechanism of spatial cognition change on aspect of embodied cognition due to high-altitude exposure.

## Introduction

Many studies have indicated that long-term exposure to high altitude may affect cognitive ability, especially the spatial information processing (Wilson et al., 2009; Nation et al., 2017). Actually, the reduction in psychological resources relative to cognitive executive control function from high-altitude exposure exhibited an obvious influence on spatial ability, which was even observed under a simulated hypoxia environment with a short-term period (Ma et al., 2016). The performance of mental rotation task, which was the ability to imagine two- or three-dimensional objects rotating in the mind (Zacks, 2008), was remarkably slower in high-altitude immigrants than that of low-altitude adults (Ma et al., 2018). Zhang et al. (2013) found that the reaction time of mental rotation task was increased in the immigrants who migrated to the high-altitude area (2,300–4,400 m) for 2 years when compared with individuals in the low-altitude area. The exposure to a simulated high-altitude (4,500 m) hypoxia environment lasting for an hour could delay the mental rotation performance (Bartholomew et al., 1999), which was corresponding to a previous finding that the reaction time was slowed down from hypoxia (Lindeis et al., 1996). Although the obvious slowness of reaction time of mental rotation from high-altitude hypoxia was observed, it is still largely unclear about the neural mechanism underlying this observation.

Electroencephalogram (EEG) with exquisite temporal resolution was a very effective tool to study the neural mechanism of mental rotation (Chen et al., 2014). Mental rotation task was first introduced by Shepard and Metzler (1971), in which the asymmetrical letters (e.g., G, J, R) were used as its normal or mirror-image version (horizontally reversed letters) in the mental rotation task (Cooper and Shepard, 1973a,b; Milivojevic et al., 2009). The reaction time linearly increases with the addition of rotation angles (Cooper and Shepard, 1973a,b; Shepard and Cooper, 1982; Eva and Hamm, 2010). The judgment of mirrored letters takes a longer time than normal letters (Cooper and Shepard, 1973a; Núñez-Peña and Aznar-Casanova, 2009). The event-related potential (ERP) study has shown that mental rotation tasks could elicit a positive ERP component between ~300 and 700 ms after stimulus presentation in the parietal region (Wijers et al., 1989); this component, known as rotation-related negativity (RRN), appeared at the parietal region ~400 ms after the presentation of a target stimulation (Heil, 2002) and was negatively correlated with the discrepancy of rotation angles (i.e., mental rotation effect; Heil, 2002; Horst et al., 2012). The mental rotation effect could be reflected on the RRN amplitude (Heil et al., 1996, 1998). The RRN amplitude under hypoxia (<15 min) was larger than that in the normoxia condition (Ma et al., 2016). The RRN amplitude in the high-altitude group related to each rotation angle condition was significantly larger than that of the low-altitude group (Ma et al., 2018). RRN is a subcluster of the P3 component, which is an indicator of the brain's cognitive resources (Ma et al., 2016). It is directly related to the brain's executive control ability under hypoxia (Ma et al., 2015a,b; Zhang et al., 2018). However, the underlying neural activity of this executive control system is still unclear.

To date, the event-related desynchronization (ERD) method has been widely used in a broad range of cognitive processes (Klimesch, 1999; Klimesch et al., 2006), such as mental rotation tasks (Klimesch et al., 2006; Chen et al., 2013) in which the motor cortical areas were involved (Bode et al., 2007). Of note, the ERD was sensitive to cognitive task performance within the alpha band (Neubauer et al., 2006; Neubauer and Fink, 2009), especially to the mental visuospatial manipulation (Williams et al., 1995). During the motor imagination task, ERD was observed in the motor cortex (Pfurtscheller and Aranibar, 1977) and was closely correlated with cortical activation reflecting a decrease in the amplitude of brain oscillatory (Pfurtscheller and Lopes da Silva, 1999), which was especially known to reflect motor cortical activity (Takemi et al., 2015). A previous study has indicated that ERD may induce cortical activity changes during motor imagery, especially the contralateral motor cortical activity during hand motor imagery (Takemi et al., 2013). An increase in the cortical activity of the spinal motoneuron was associated with ERD magnitude during hand motor imagery (Takemi et al., 2015). The motor imagery–induced ERD was usually observed within the alpha and beta bands, which frequently served as a neural marker to characterize the activation of sensorimotor cortex (Aono et al., 2013). Compared with the only upright hand pictures, the hand pictures presented with different orientations took a longer reaction time during the hand mental rotation task, which elicited the stronger alpha ERD and beta ERD (Yu et al., 2020). During the mental rotation task, ERD could be usually observed in the central–parietal region within the alpha and beta bands at ~300–800 ms (Riečanský and Katina, 2010; Yan et al., 2012; Lyu et al., 2016; Wang et al., 2016); it has also been found to be correlated with the reaction time (Riečanský and Katina, 2010). The alpha power and beta power were negatively correlated with reaction time at ~400 ms in the parietal region; it means that the faster reaction time was associated with the smaller ERD, whereas the slower reaction time was with the larger ERD (Riečanský and Katina, 2010). Of note, the central alpha ERD was correlated with mental rotation processing, in which the alpha ERD was increased with the slower reaction time (Chen et al., 2013). Beta ERD was related to the rotation angles at the later stage of the mental rotation task (Ozga et al., 2018). As to the parietal region, previous studies have already confirmed that it is a target region to be significantly activated during the mental rotation task (Alivisatos and Petrides, 1997; Harris and Miniussi, 2003). So far, there was consistent evidence pointing to that the parietal region was the core cortex to be related to the spatial transformation of mental rotation (Collins and Kimura, 1997; Save and Poucet, 2000; Jagaroo, 2004). Moreover, hypoxia exposure affected the activity of the parietal region related to mental rotation (Seurinck et al., 2004; Wilson et al., 2009). And the larger bilateral parietal regions were observed to be activated under hypoxia condition (Ma et al., 2016). That is to say, ERD can be used as another imaging indicator to reveal the neural basis of hypoxia affecting mental rotation. Given that mental rotation involves a combination of cognitive processing and sensorimotor abilities, ERD, which is different from RRN, which reflects cognitive executive control, helps understanding in depth the effect of hypoxia on brain function.

Based on the consideration mentioned previously, the present study aimed to explore cortical activity related to mental rotation in high-altitude immigrants using the mental rotation task combined with ERD, EEG time–frequency analysis, and source localization. To this end, this study recruited healthy young participants who were born and raised up to the early adulthood in the low-altitude areas but then immigrated to high-altitude areas and had been living in Tibet (~3,680 m) for 3 years and those who were born and lived at sea level in Guangzhou, China. We explored the differences of ERD underlying the mental rotation task and the current source density distribution across cerebral cortex to show the neural mechanism of the mental rotation in these immigrants, when compared to those of the normal control group.

## Materials and Methods

### Participants

The present study recruited a total of 32 healthy immigrants (aged 19–22 years) in Tibet University; all of them were born and grew up to early adulthood in the low-altitude areas (<1,500 m) and had migrated to the high-altitude area (Lhasa, 3,680 m) for 3 years. In addition, we also recruited a total of 33 healthy college students (aged 19–22 years) in South China Normal University in Guangzhou (the average altitude of 11 m) as the normal control group. The participants in the normal control group were natives of low-altitude regions who never visited high altitudes (>1,500 m). The two groups were matched on gender (high altitude: 15 male, 17 female; low altitude: 17 male, 16 female; *p* = 0.82), the college entrance examination scores (high altitude: 509.55 ± 26.78; low altitude: 509.76 ± 109.55; *p* = 0.10), and intelligence scores (Raven Progress Matrices; high altitude: 36.10 ± 5.10; low altitude: 41.81 ± 6.52; *p* = 0.09). There was no significant difference between the two groups in their intelligence scores on the Raven Intelligence Test (*p* > 0.05). All of them were Han nationality and right-handed; none of these participants reported a history of neurological or psychiatric disorders. They were required not to drink alcohol or caffeinated beverages within 24 h before the experiment. All participants had normal or corrected-to-normal vision. All participants were compensated for participation and provided written informed consent prior to the experiment.

### Materials

**Figure 1:**
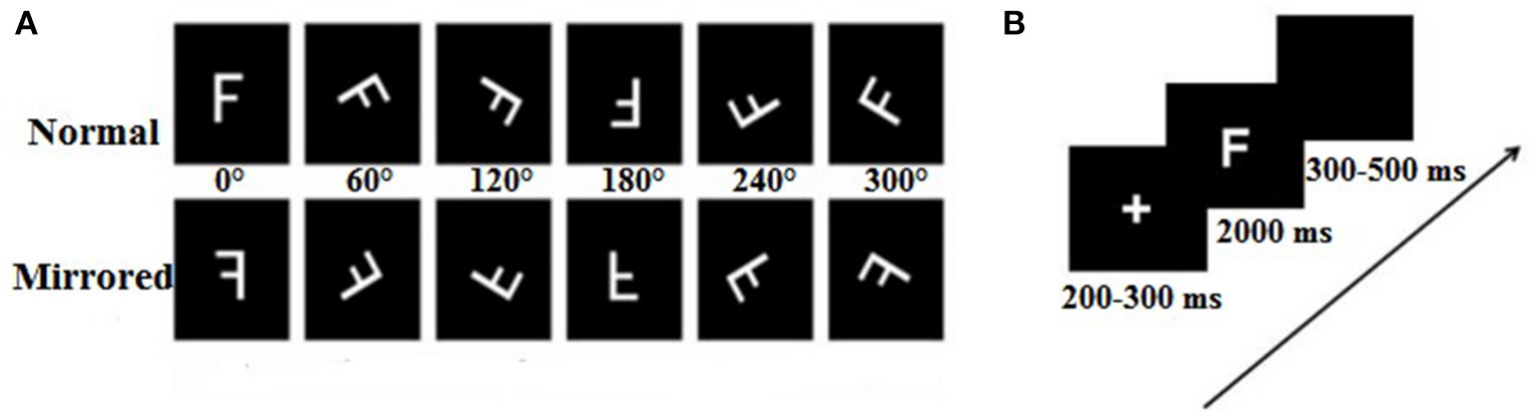
Samples of experimental stimuli and experimental procedure. **(A)** The normal and mirrored versions of the F letters in each orientation (from 0 to 300°). **(B)** The experimental procedure.

Six capital letters (F, P, R, L, G, and Q) were presented independently either in its normal version or in mirror image, rotated six rotation angles (0, 60, 120, 180, 240, and 300°) either clockwise or counterclockwise from the upright position (Figure 1A). The stimulus materials were completely consistent with a previous study (Ma et al., 2016).

### Procedure

The stimulus presentation and behavior data collection were completed by using *E*-prime software (version 2.0). The participants were seated in a dim light, electrically shielded, and a sound-attenuating room facing the computer screen. The stimuli were displayed in white on a black background at the center of computer screen, at a distance of ~75 cm from participants' eyes, resulting in a vertical visual angle of 4.58° and horizontal visual angle of 3.05°. The laboratory experimental setting and equipment were the same between Tibet University and South China Normal University. Each trial began with the presentation of a fixation point in the center of a computer monitor, which was shown for a random duration between 200 and 300 ms, followed by the letter with a 2,000 ms presentation time. The participants were instructed to judge whether the letter was normal or mirrored by keystroke reaction (F: normal letters, J: mirrored letters). If no response was made within 2,000 ms, the stimulus would automatically disappear from the screen, and the trial was encoded as “no response.” Finally, a black screen appeared lasting for 300–500 ms before the next trial. The participants were asked to respond as quickly as possible while keeping feedback errors to a minimum. In addition, the participants were asked not to make eye movements or blink during trials, but to consistently blink several times immediately after a response. Reaction time and judgments (F/J) were recorded on the stimulus-control computer. At the beginning of the session, the participants completed a practice block of 12 trials to be familiar with the experimental process and achieved at least 90% accuracy rate in order to enter the formal experiment. Each participant needed to complete five blocks, and they could have a rest between blocks. The number of task-related variables (stimulus types, angle) was randomly presented. In each block, there was a total of 72 trials with the following variables consisting of six rotation angles (0, 60, 120, 180, 240, and 300°), stimulus types (normal and mirrored letters), and stimulus letters (F, P, Q, G, L, and R) presented one time; six stimulus letters were used equally under all conditions. Finally, the whole experiment lasted ~1–1.5 h, from wearing the electrode cap to finishing the experiment. The experimental procedure is shown in Figure 1B.

### EEG Data Acquisition

According to the 10–20 International System, the EEG data were recorded from 64-channel Ag/AgCl electrodes placed on the scalp (Curry7; http://compumedicsneuroscan.com/products-overview/). Participants washed and blew their hair dry before the experiment. The left and the right mastoids (M1 and M2 electrodes) and a ground electrode on the medial aspect, which was ~2 cm posterior to CZ, were used as reference electrodes. All interelectrode impedance was kept below 5 kΩ, and the sampling rate of the amplifier was 500 Hz. Two electrodes were placed above and below the left eye about 1 cm from the left vs. right orbital rim, used to record the electro-oculogram (EOG) data. One of the electrodes was used to monitor horizontal eye movement (HEOG), and the other one was used to monitor vertical eye movement (VEOG). The EEG data were offline re-referenced to the average data of the left and the right mastoids, using the 30 Hz low-pass filter and 0.5 Hz high-pass filter to remove line noise and baseline drift, respectively. The sampling rate of EEG signal was reduced from 500 to 256 Hz, filtered digitally offline with a 0.1- to 30-Hz bandpass filter; the EEGs contaminated with artifacts containing EOG, electrocardiogram, and electromyography (artifacts with amplitude exceeding 80 μV) were manually deleted. Independent component analysis was completed in EEGLAB to remove artifacts in data components, such as eye movements, blinks, and muscle tension. It was important to note that only the trials with correct response in behavioral experiment were included in EEG data analysis. Each participant was asked to complete 360 trials. The averaged epoch for each trial was 1,000 ms, beginning 200 ms before and ending 800 ms after the stimulus presentation, with 200 ms of each epoch corresponding to a prestimulus baseline.

For the determination of participant-specific frequency bands, the comparison of two short-time power spectra was used, which was calculated by averaging over a great quantity of event-related EEG trials (Pfurtscheller and Lopes da Silva, 1999). The percentage decrease (or increase) from the reference interval (R) (while the fixation cross was shown) to the activation interval (A) (before responding) was defined as ERD% = [(A – R)/R] × 100% (Pfurtscheller, 2001). The quantification of ERD/event-related synchronization (ERS) was divided into four steps, firstly, the bandpass filtering was carried out for all event-related trials; second, the amplitude samples were squared to obtain the power samples; once again, the power samples of all trials were averaged; eventually, the time samples were averaged to make the data smooth and reduce variability (Pfurtscheller and Lopes da Silva, 1999).

### Data Analysis

#### Behavioral Data

Our primary behavioral measure was reaction time and error rate. Reaction time was analyzed using a 2 × 2 × 6 mixed analysis of variance (ANOVA), with group (high-altitude and low-altitude) as a between-subject factor, taking stimulus types (normal and mirror-reversed) and rotation angles (0, 60, 120, 180, 240, and 300°) as within-subject factors.

#### EEG Data Analysis

The time–frequency analysis of (FZ, FCZ, CZ, CPZ, and PZ) channels at five electrodes was carried out and operated by MATLAB and Fieldtrip toolbox. In the time window beginning 200 ms before and ending 800 ms after the stimulus presentation, there was no overlap between the time windows. The significant differences between conditions within frequency bands and at time windows were first determined and then derived the mean value of data energy in the corresponding ranges for statistical analysis. We analyzed the data from five electrodes with all angles. The alpha ERD and the beta ERD at multiple time windows were analyzed, respectively, using a 2 × 2 × 6 × 5 mixed-design repeated-measure ANOVA, with the groups as a between-participants factor, taking stimulus types, angles, and electrodes (FZ, FCZ, CZ, CPZ, and PZ) as within-participant factors. Through using the Greenhouse–Geisser method to correct the *p*-value when the statistical results were not spherical, the multiple comparisons were corrected by the Bonferroni method.

#### Current Source Density Analysis

Standard low-resolution brain electromagnetic tomography (sLORETA) software was applied to spatially identify and analyze the source location of cortical activity through using the traditional EEG recordings (Pascual-Marqui et al., 2002). The statistical nonparametric mapping in sLORETA was used to analyze source current density across cortical regions. Group differences were assessed using independent-samples *t*-tests evaluated at *p* < 0.01 corrected. Statistical correction for multiple comparisons was performed using *t*-values to characterize the difference of the standardized current density values between the two groups. The significant mental reaction effect on reaction time was the longest for upside-down orientation (180°; Ma et al., 2018). The sLORETA was used for locating the source of the cortical activity underlying mental rotation task for the 180° stimulus condition between two groups.

## Results

### Behavioral Results

**Figure 2:**
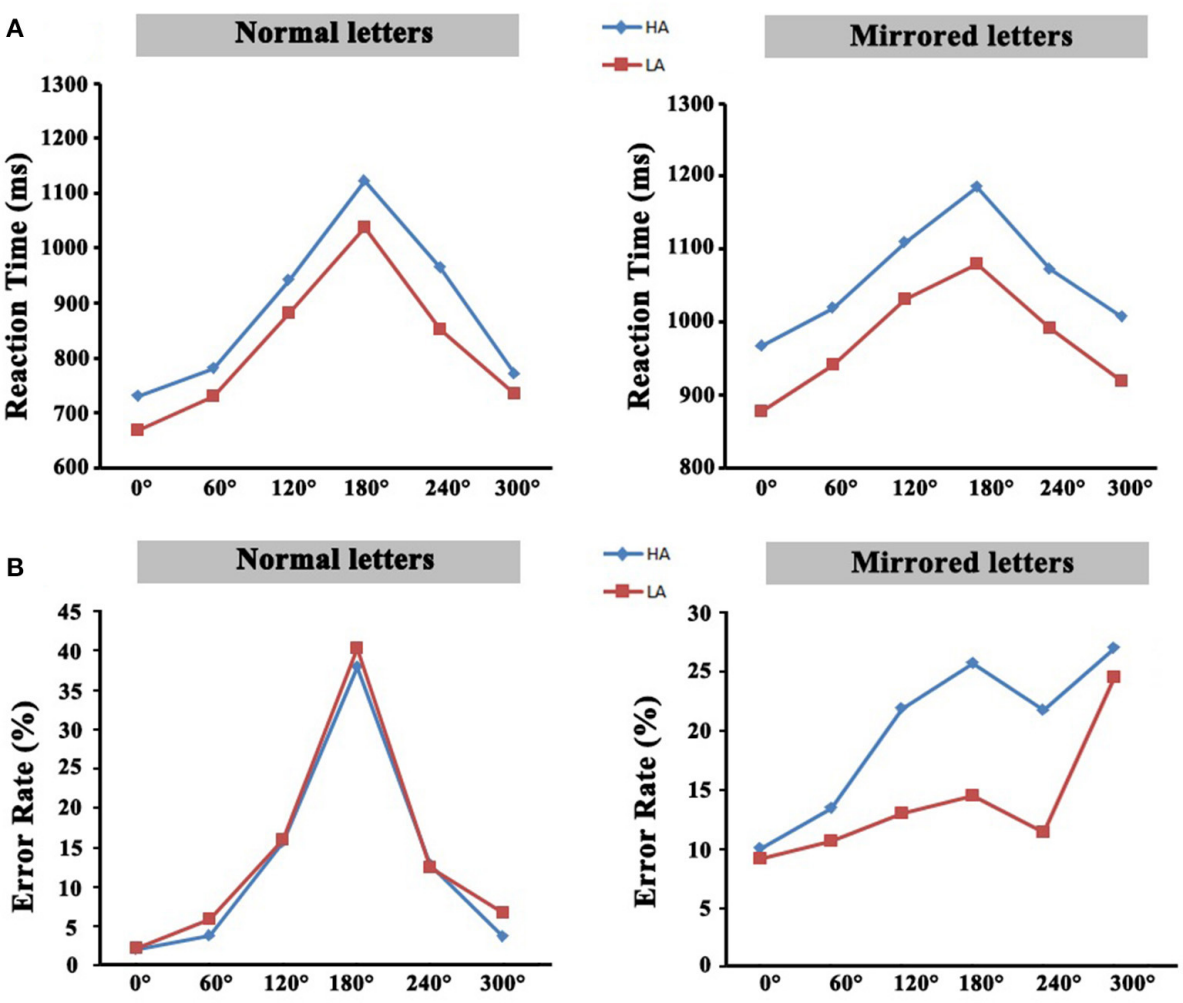
**(A)** The average reaction time of all rotation angles. **(B)** The average error rate of all rotation angles. HA, high-altitude; LA, low-altitude.

The analysis of reaction time revealed that there was a significant mental rotation effect in the high-altitude group and the low-altitude group. The reaction time of the high-altitude group was significantly slower than that of the low-altitude group. These mental rotation effects derived from reaction time were also observed on aspect of error rates in the two groups. All these results are depicted in Figure 2. The details of the behavior results could also be found in a previous study (Ma et al., 2018).

### EEG Results

#### Time–Frequency Spectrum Power Results

**Figure 3:**
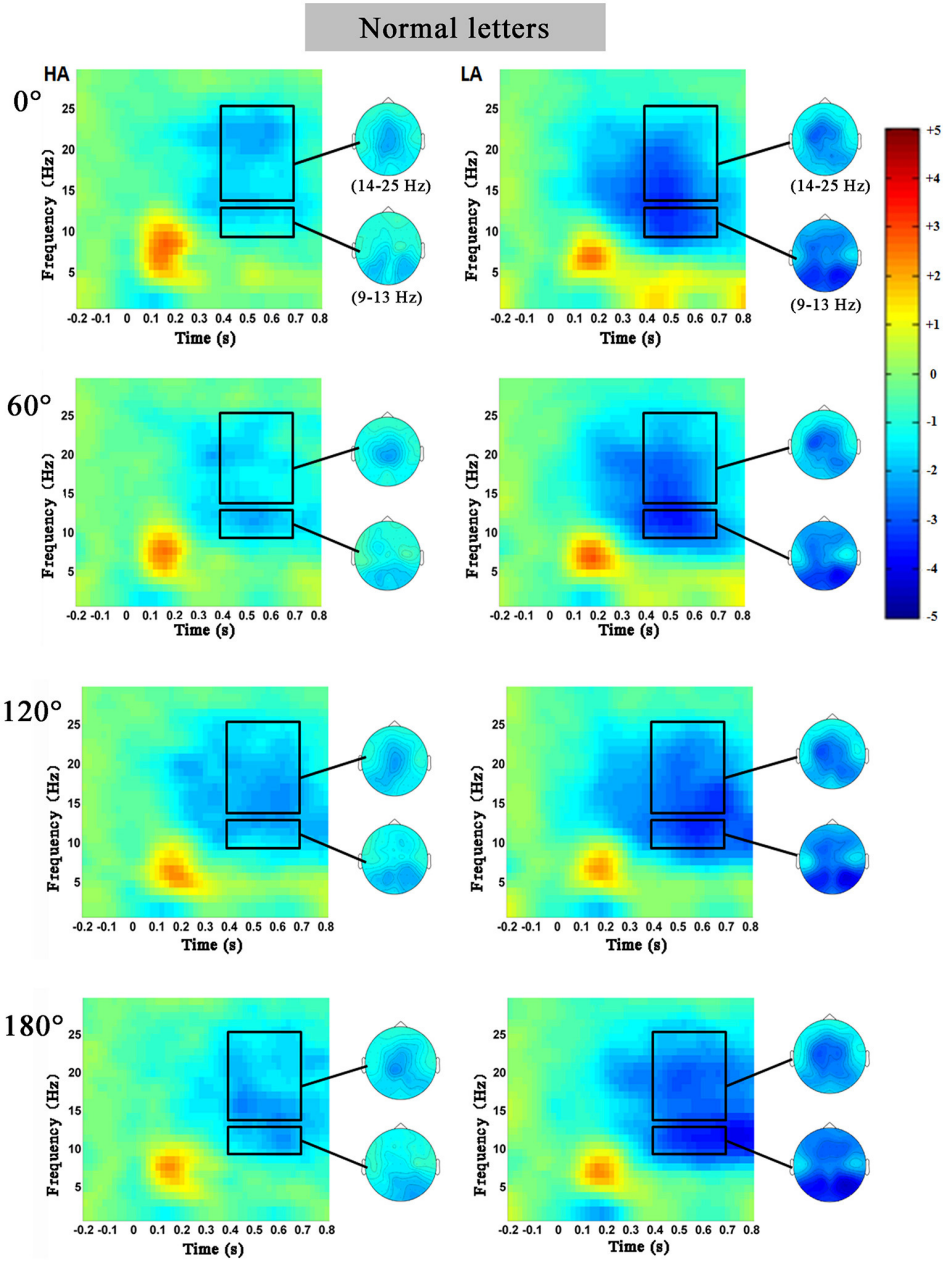
The average time–frequency spectrum power on PZ electrode at the normal letters for the high-altitude (HA) and the low-altitude (LA) groups during the mental rotation task. The black rectangle was the oscillation power in the alpha and beta bands, and on the right was the corresponding topographical map.

**Figure 4:**
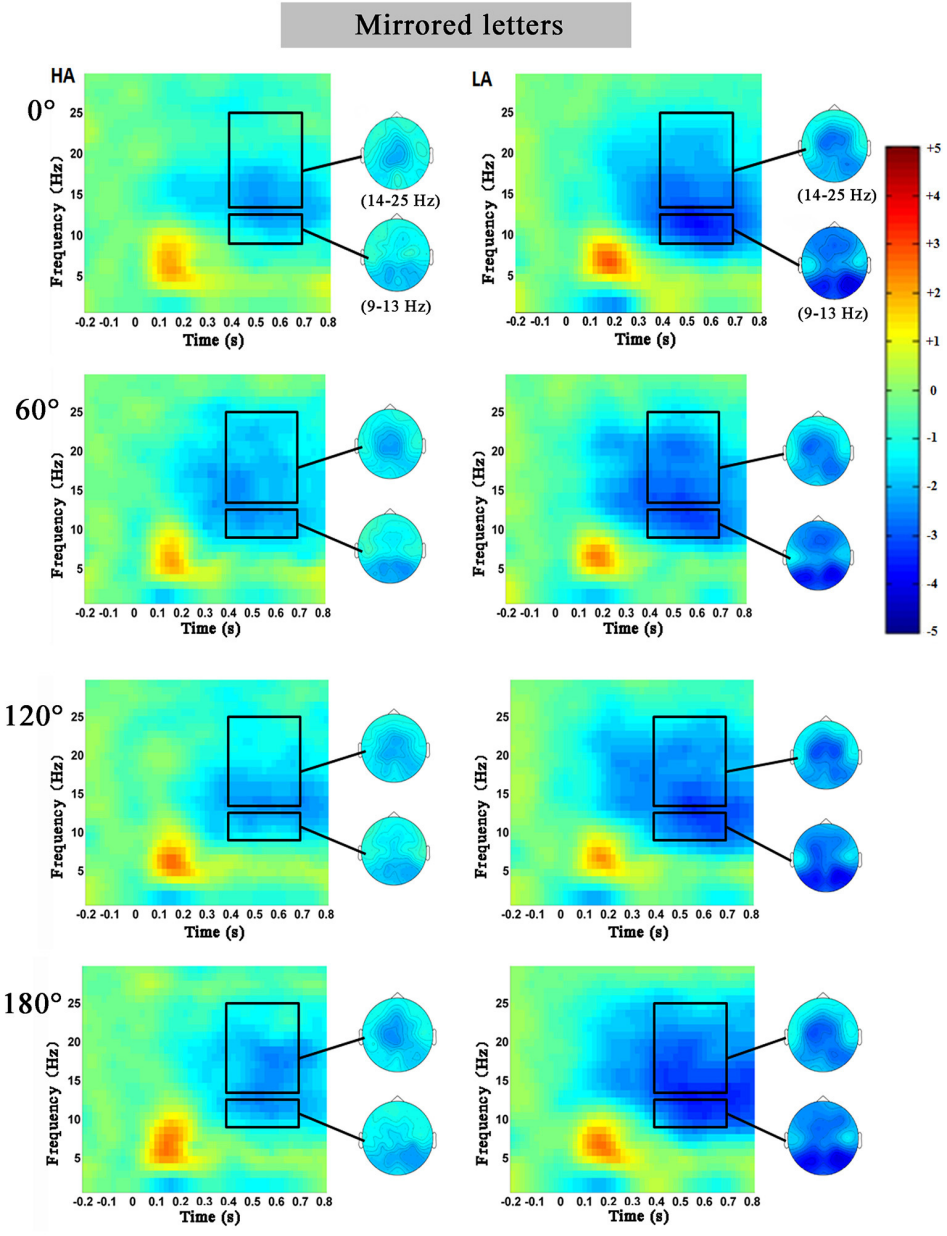
The average time–frequency spectrum power on PZ electrode at the mirrored letters for the high-altitude (HA) and the low-altitude (LA) groups during the mental rotation task. The black rectangle was the oscillation power in the alpha and beta bands, and on the right was the corresponding topographical map.

Figures 3, 4 showed the characteristics of time–frequency spectrum power on PZ electrode and all angles at the normal letters and mirrored letters in the two groups. The results showed that PZ electrode with 0–180° stimulus condition obviously existed difference. The alpha ERD and the beta ERD (the blue part circled with a black frame) were obviously observed in 400–700 ms after the target stimulation presented during the mental rotation task. Compared with the low-altitude group, ERD was obviously decreased in the high-altitude group; this observation was the same in the other four electrodes (FZ, FCZ, CZ, and CPZ) and all angles. In order to more objectively show the differences in the mental rotation task, the oscillation power (black frame) in the alpha and beta bands was further extracted for statistical analysis.

##### Alpha Band

**Figure 5:**
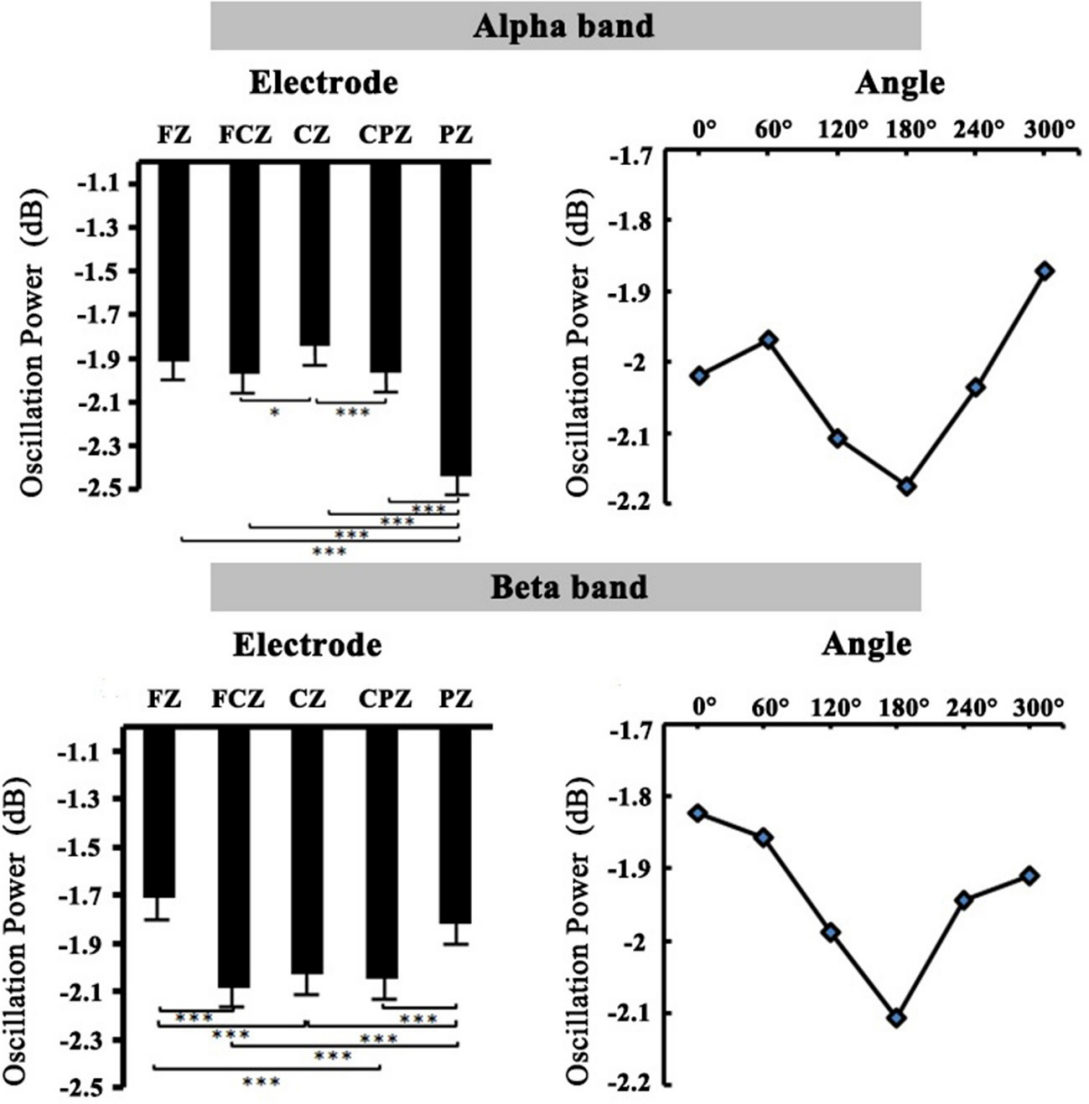
The relation diagram of electrodes, angles, and oscillation power in the alpha and beta bands.

The 2 (group: high-altitude and low-altitude) × 2 (stimulus types: normal and mirror-reversed) × 6 (angle: 0, 60, 120, 180, 240, and 300°) × 5 (electrode: PZ, CPZ, FCZ, FZ, and CZ) repeated-measure AVOVA was applied in this study. The statistical analysis in the 400–700 ms time window supported the observations in the alpha band (9–13 Hz). The results showed that the main effect of angles was significant [*F*_(5,315)_ = 2.28, *p* = 0.047, η^2^ = 0.007]; the alpha power at 60° (*p* = 0.042) and that at 300° (*p* = 0.002) were significantly higher than that at 180°, respectively (Figure 5). The main effect of stimulus types was not significant [*F*_(1,63)_ = 1.017, *p* = 0.314, η^2^ = 0.03]. Simple effect analysis showed that the alpha power of the high-altitude group was significantly higher than that of the low-altitude group [FZ: *F*_(4,252)_ = 99.249, *p* < 0.01; FCZ: *F*_(4,252)_ = 65.303, *p* < 0.01; CZ: *F*_(4,252)_ = 53.736, *p* < 0.001; CPZ: *F*_(4,252)_ = 42.872, *p* < 0.01; PZ: *F*_(4,252)_ = 67.95, *p* < 0.001].

##### Beta Band

The 2 (group: high-altitude and low-altitude) × 2 (stimulus types: normal and mirror-reversed) × 6 (angle: 0, 60, 120, 180, 240, and 300°) × 5 (electrode: PZ, CPZ, FCZ, FZ, and CZ) repeated-measure AVOVA was applied to data analysis in the beta band (14–25 Hz). The statistical analysis in the 400–700 ms time window showed that the main effect of stimulus types was significant [*F*_(1,63)_ = 13.841, *p* < 0.001, η^2^ = 0.041]; the beta power of normal letters (−1.866 ± 0.055 dB) was significantly higher than that of mirrored letters (−2.01 ± 0.052 dB). The main effect of angles was significant [*F*_(5,315)_ = 5.01, *p* < 0.001, η^2^ = 0.015], which found that the beta power at 0, 60, 240, and 300° was significantly higher than that at 180°, respectively (all *p* < 0.05; Figure 5). Simple effect analysis showed that the beta power of the high-altitude group was significantly higher than that of the low-altitude group [FZ: *F*_(4,252)_ = 89.353, *p* < 0.01; FCZ: *F*_(4,252)_ = 74.517, *p* < 0.01; CZ: *F*_(4,252)_ = 64.914, *p* < 0.001; CPZ: *F*_(4,252)_ = 56.679, *p* < 0.001; PZ: *F*_(4,252)_ = 52.443, *p* < 0.01].

**Figure 6:**
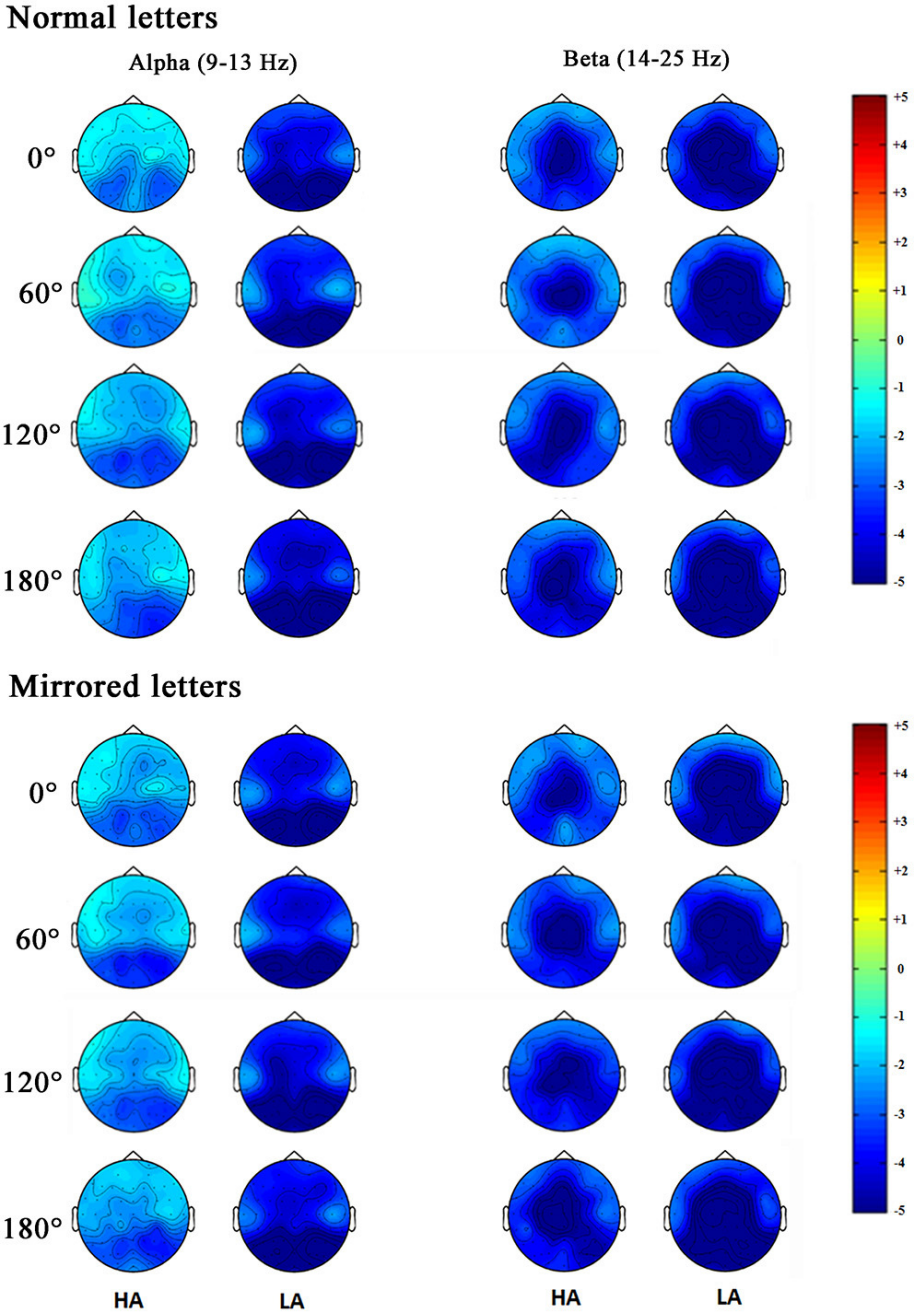
Topographical distribution of the alpha and beta bands power were respectively measured in the 400–700 ms time window during the mental rotation task at the normal letters and mirrored letters.

In order to observe the spatial distribution characteristics of ERD, we obtained the topographical distribution of the alpha and beta bands power in the 400–700-ms time window during the mental rotation task (Figure 6). The ERD phenomenon in the high-altitude group was decreased; the high-altitude group elicited the smaller alpha ERD and beta ERD. Alpha ERD was mainly observed in the parietal–occipital regions, and beta ERD was mainly in the central–parietal regions.

#### Current Source Density Results

**Figure 7:**
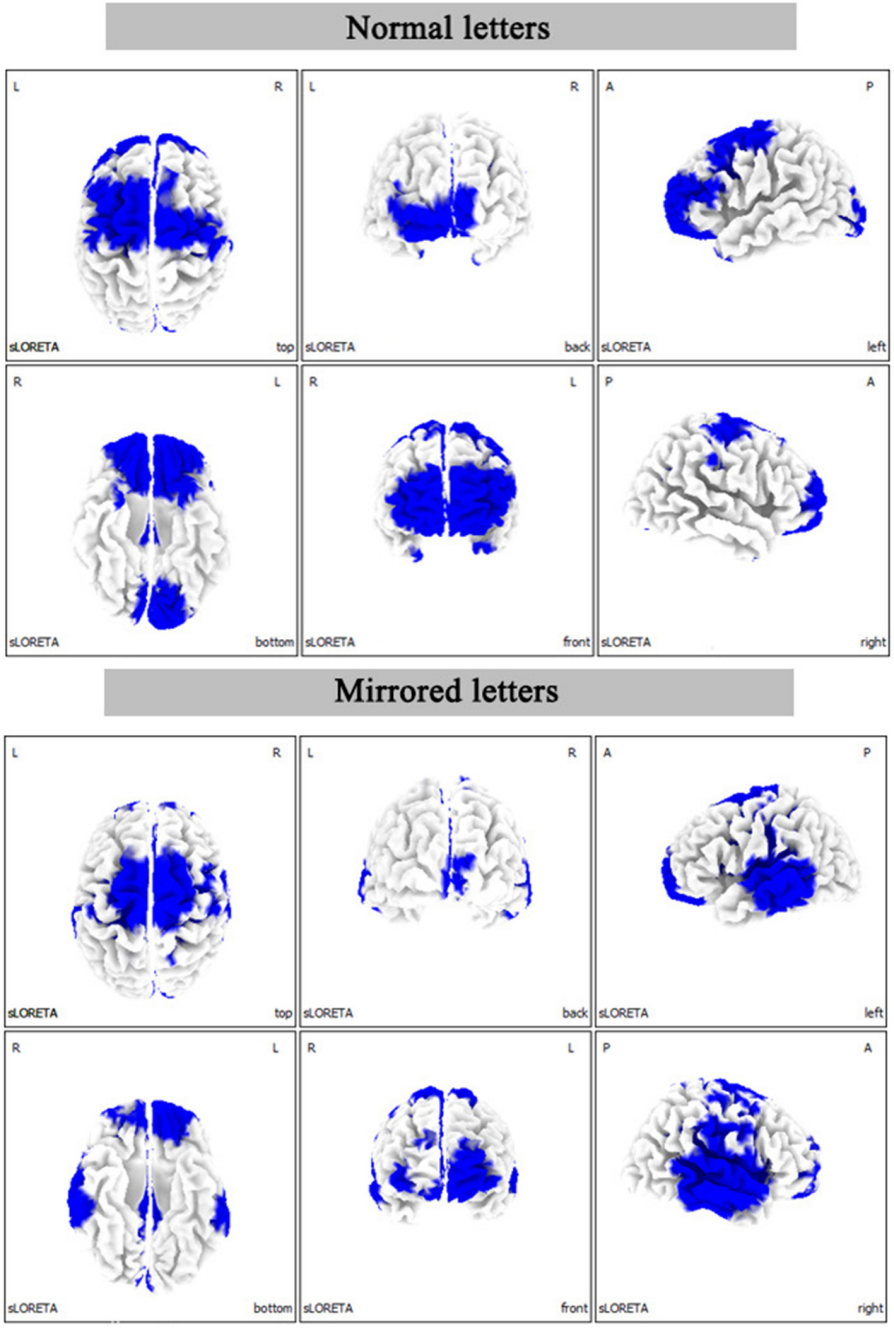
The statistical differences between the high-altitude and the low-altitude groups in the three-dimensional map of cerebral cortex distribution during the mental rotation (180°) task. L, left; R, right; A, anterior; P, posterior.

The statistical results of EEG activity in the Brodmann area at normal or mirrored letters and brain regions where significant group differences were found are shown in Tables 1, 2. As seen in the Tables 1, 2, the black frame represented the same brain regions with significant group differences between the normal letters and the mirrored letters, involving the frontal lobe, the parietal lobe, the occipital lobe, the temporal lobe, and the limbic lobe. Voxels in which the high-altitude group showed reduced current source density as compared to the low-altitude group were projected onto a surface model of the brain (Figure 7), suggesting that the cortical activity was significantly decreased during mental rotation task under the hypoxia environment.

**Table 1:** Statistical comparison of current source density at the normal letters between the high-altitude and the low-altitude groups during the mental rotation task (*t*-value from highest to lowest).

	**Region**	**Brodmann area**	***t-*value**
Normal letters	Limbic lobe	32	−6.05
	Frontal lobe	10	−6.02
	Frontal lobe	11	−5.99
	Limbic lobe	24	−5.78
	Frontal lobe	25	−5.77
	Frontal lobe	47	−5.67
	Parietal lobe	3	−5.64
	Frontal lobe	6	−5.59
	Frontal lobe	9	−5.59
	Occipital lobe	18	−5.58
	Frontal lobe	4	−5.56
	Parietal lobe	2	−5.53
	Occipital lobe	17	−5.53
	Limbic lobe	31	−5.47
	Limbic lobe	23	−5.37
	Frontal lobe	46	−5.28
	Frontal lobe	8	−5.20
	Parietal lobe	1	−5.14
	Occipital lobe	30	−5.13
	Temporal lobe	38	−5.12
	Frontal lobe	45	−5.11
	Temporal lobe	39	−5.10
	Occipital lobe	19	−5.05

**Table 2:** Statistical comparison of current source density at the mirrored letters between the high-altitude and the low-altitude groups during the mental rotation task (*t*-value from highest to lowest).

	**Region**	**Brodmann area**	***t-*value**
Mirrored letters	Parietal lobe	2	−6.80
	Parietal lobe	3	−6.79
	Frontal lobe	4	−6.79
	Frontal lobe	6	−6.70
	Limbic lobe	24	−6.49
	Limbic lobe	31	−6.44
	Parietal lobe	40	−6.39
	Temporal lobe	22	−6.16
	Temporal lobe	41	−6.11
	Temporal lobe	42	−6.09
	Limbic lobe	23	−6.01
	Temporal lobe	21	−5.77
	Frontal lobe	43	−5.72
	Frontal lobe	9	−5.72
	Limbic lobe	32	−5.70
	Frontal lobe	44	−5.69
	Limbic lobe	33	−5.60
	Temporal lobe	13	−5.56
	occipital lobe	18	−5.12
	Parietal lobe	7	−5.11
	Frontal lobe	11	−5.10
	Temporal lobe	38	−5.10
	Frontal lobe	10	−5.09
	Frontal lobe	5	−5.08

## Discussion

This study explored the cortical activity on the aspect of ERD underlying the mental rotation task between the high-altitude and the low-altitude groups based on EEG technology. The main findings can be summarized as follows: (1) the high-altitude hypoxia significantly slowed the mental rotation reaction; (2) the decreased ERD was correlated with the reduction of sensorimotor-related cortical activity during the mental rotation task from hypoxia exposure; (3) the weakening of alpha ERD and that of beta ERD were found in the 400–700 ms time window from hypoxia exposure.

In terms of behavior performance, there was an obvious mental rotation effect that the judgment time of rotation stimulation increased with the increase in rotation angles in proportion to approximately linear mode, which was consistent with the previous research results (Jordan et al., 2001; Milivojevic et al., 2003). As the rotation angles increase, the high-altitude immigrants had slower reaction times and fewer correct responses responding to the mental rotation task, which might reveal the reduction related to the spatial cognition in the high-altitude group. The two groups had no significant difference on normal letters in the error rate, but the error rate in the high-altitude group was significantly higher than that of the low-altitude group on mirror letters.

The oscillation power of alpha and beta bands was correspondingly inhibited with the increase in rotation angles in the mental rotation task (Figure 5). Of note, the present study found that ERD was detected in the motor-related cortex. ERD has been reported to be an electrophysiological index of the activation of cortical region (Pfurtscheller, 2001). There was a reliable evidence that ERD was used as an index to measure cortical activity in the process of motor imagination or real movement (Pfurtscheller et al., 2006), as well as to be associated with activated motor networks (Pfurtscheller et al., 2008). Through using the time–frequency analysis, the present study further demonstrated that the alpha ERD and beta ERD were decreased in the high-altitude group during 400–700 ms time window, proving that the cortical activity was decreased from high-altitude exposure (Cummins et al., 1993). This observation was corresponding to a previous finding that the inhibited cortex activities from hypoxia exposure were primarily observed in motor cortical activity (Neubauer and Sunderram, 2004; Marillier et al., 2017). To sum up, these findings might collectively confirm that high-altitude exposure led to a decreased cortical activity.

Moreover, the alpha ERD and beta ERD recorded on FZ, FCZ, CZ, CPZ, and PZ electrodes in the mental rotation task confirmed that the letter rotation was indeed involved in the processing of motion imagination. Analyzing the topographical distribution map (Figure 6) demonstrated that the decreased alpha ERD was mainly observed in the parietal–occipital regions, and the decreased beta ERD was mainly observed in the central–parietal regions. Previous studies had observed that the alpha ERD was mainly related to the primary sensory processing (Schürmann et al., 1997; Kolev et al., 1999; Schürmann and Başar, 2001), and the greater alpha ERD was observed during the larger of cognitive loads (Peng et al., 2012). While the beta ERD was related to cognitive control function, which was mainly located in the primary motor cortex (Muthukumaraswamy and Johnson, 2004). The beta ERD was associated with a real or an imagined movement, especially with motor planning (Tzagarakis et al., 2010) and a sensorimotor activity (Bechtold et al., 2018). Combined with the results of behavior performances, it took a longer time to complete the mental rotation task in the high-altitude group, which was associated with the attenuation of the alpha ERD and the beta ERD. The decreased alpha ERD was found to reflect the active information processing (Klimesch et al., 2007), and the reduced beta ERD was closely linked with the motor processes and motor imagery (Nam et al., 2011). The cortical activity during motor imagery was associated with the ERD magnitude (Takemi et al., 2013). The delay of mental rotation reaction from hypoxia exposure may be associated with the decreased alpha ERD and beta ERD. During the mental rotation task, the alpha ERD and beta ERD in sensorimotor regions were considered to be related to cortical activity in sensorimotor networks (Chen et al., 2013; Lyu et al., 2016). The sensorimotor cortical activity would be rapidly decreased during acute hypoxia (Ruggiero et al., 2018). Correspondingly, our results suggested that the sensorimotor cortical activity was reduced and was inhibited during the mental rotation task in the high-altitude group; this may be a compensated mechanism for adaptation to hypoxic exposure environment.

During movement observation, the ERD level was reduced in the patients with Parkinson disease for observed hand actions in the alpha and beta band (Heida et al., 2014). ERD may reveal abnormal characteristics of the associated neural system during mental rotation tasks in hypoxia exposure. Recently, a lot of researchers have demonstrated that the beta oscillation in the frontal–central area was related to cognitive control processing, which was considered to be an effective physiological index of response control processing (Aron, 2011; Huster et al., 2013). The beta ERD was decreased in the high-altitude group, suggesting that high-altitude immigrants needed a longer response time to complete the mental rotation task, which may be associated with the impaired control function. The beta ERD in the premotor cortex with the increase in rotation angles might be related to using motor strategies during the mental rotation task (Ozga et al., 2018). We speculated that using the motor imagination strategy to complete mental rotation task was able to consume more mental resources, and there was a significant difference in beta power between the two groups, which indicated that the high-altitude group needed more cognitive control processing ability. Therefore, the cortical activity was associated with the ERD magnitudes from the long-term hypoxia exposure, which may be related to the decrease in sensorimotor cortical activity.

Many previous studies have shown that there was a significant reaction slowness from the high-altitude exposure, in which there was reduced cognitive control function, which was derived from the reduced cognitive resources in the high-altitude areas (Wang et al., 2014; Ma et al., 2019). To effectively supplement this discovery of the predecessors, this study has determined that the cortical activity of the sensorimotor area is related to the decline of motor operation ability by using specific spatial manipulation tasks, and this phenomenon may reflect the reduction of cognitive control ability.

Importantly, the reduction of sensorimotor cortical activity related to ERD might be involved in cognitive control function. In high-altitude immigrants with long-term hypoxia exposure, the present finding provided new insights into the relationship between sensorimotor cortical activity and cognitive control function, which might be a new direction to understand the neural mechanism of aging and neurodegenerative diseases, especially Parkinson disease and others involving decreased sensorimotor ability. According to classical cognitive science, cognition is something after perception. Therefore, the slower motor response is separated from the decreased cognition, and the decline of cognitive resources caused by altitude exposure independently affects cognitive control ability and motor response ability. On the contrary, according to the view of embodied cognition, cognitive ability was rooted in sensorimotor ability (Bechtold et al., 2018; Naro et al., 2021). According to this, the lack of cognitive resources will be reflected in executive control ability and sensorimotor ability at the same time. This study used the mental rotation task, which integrates cognitive load and sensorimotor ability at the same time, and found a close connection between executive control ability and sensorimotor ability. As we all know, mental rotation task is a classic paradigm of cognitive science, which provided an important experimental evidence to confirm the existence of mental imagery as an independent information representation format in cognitive science. Nowadays, the ending of mental imagery debate has established an independent means of mental imagery (Pearson and Kosslyn, 2015). Thus, revealing the cognitive neural process behind the mental rotation task that reflects embodied cognitive perspectives has become a new perspective for studying brain functions. From the perspective of hypoxia, this study confirmed the neural mechanism that reflects the mental rotation task that reflects the embodied cognitive perspective and discovered the role of sensorimotor cortex in executive control, providing a new perspective for follow-up research.

The limitation of our study is not a direct measurement of neuronal excitability, but an indirect measurement. Previous studies have shown that living in a high-altitude area causes a reduced partial pressure of inspired oxygen and lower hemoglobin oxygen saturation, which can lead to hypoxia (Beall, 2000; Virués-Ortega et al., 2011). Oxygen saturation decreased on long-term high-altitude exposure (Ma et al., 2015b), and the oxygen saturation levels decrease with acute altitude (Dünnwald et al., 2021). A study of Andean natives using positron emission tomography (PET) scans showed that the lower region-by-region brain metabolic rates may be a defense mechanism against chronic hypoxia at high altitude (Hochachka et al., 1994). Through using the blood oxygen level–dependent technique with functional magnetic resonance imaging (fMRI), the study showed that compared with short-term hypoxic exposure, long-term hypoxia exposure reduced cerebral deoxy hemoglobin balance (Rostrup et al., 2005). Therefore, using PET, MRI, or other hematological measurements will be the future direction to deepen understanding of the relative topic.

## Conclusion

This study revealed the dynamic process of mental rotation task under hypoxia environment from both behavior responses and ERD of EEG signals. The results of time-frequency analysis showed that there was existing decreased alpha ERD and beta ERD in the 400–700 ms time window during the mental rotation task in the high-altitude group. Mental rotation delay from hypoxic exposure may be related to the weakening of alpha ERD and beta ERD, which embody the reduced sensorimotor-related cortical activity relative to cognitive control function. The present findings showed the potential association of sensorimotor-related cortical activity with cognitive control, which provided new insights into the neural mechanism of the spatial manipulation change on embodied cognition in high-altitude immigrants.

## Data Availability Statement

The raw data supporting the conclusions of this article will be made available by the authors, without undue reservation.

## Ethics Statement

The studies involving human participants were reviewed and approved by The Ethics Committee of Tibet University. The patients/participants provided their written informed consent to participate in this study.

## Author Contributions

D-lZ and H-lM designed the experiment and collected the data. Z-qX, Y-lH, and G-lL analyzed the data. D-lZ and Y-lH prepared the manuscript. All authors contributed to the article and approved the submitted version.

## Conflict of Interest

The authors declare that the research was conducted in the absence of any commercial or financial relationships that could be construed as a potential conflict of interest.

## References

[B1] Alivisatos B. Petrides M. (1997). Functional activation of the human brain during mental rotation. Neuropsychologia 35, 111–118. 10.1016/S0028-3932(96)00083-8 9025115

[B2] Aono K. Miyashita S. Fujiwara Y. Kodama M. Ushiba J. (2013). Relationship between event-related desynchronization and cortical excitability in healthy subjects and stroke patients. Tokai J. Exp. Clin. Med. 38, 123–128. 24318283

[B3] Aron A. R. (2011). From reactive to proactive and selective control: developing a richer model for stopping in appropriate responses. Biol. Psychiatry 69, e55–e68. 10.1016/j.biopsych.2010.07.024 20932513 PMC3039712

[B4] Bartholomew C. J. Jensen W. Petros T. V. Ferraro F. R. Fire K. M. Biberdorf D.. (1999). The effect of moderate levels of simulated altitude on sustained cognitive performance. Int. J. Aviat. Psychol. 9, 351–359. 10.1207/s15327108ijap0904_3 11543214

[B5] Beall C. M. (2000). Tibetan and andean patterns of adaptation to high-altitude hypoxia. Hum. Biol. 72, 201–228. 10721618

[B6] Bechtold L. Ghio M. Lange J. Bellebaum C. (2018). Event-related desynchronization of mu and beta oscillations during the processing of novel tool names. Brain Lang. 177–178, 44–55. 10.1016/j.bandl.2018.01.004 29421271

[B7] Bode S. Koeneke S. Jäncke L. (2007). Different strategies do not moderate primary motor cortex involvement in mental rotation: a TMS study. Behav. Brain Funct. 3:38. 10.1186/1744-9081-3-38 17683644 PMC1994952

[B8] Chen X. Bin G. Daly I. Gao X. (2013). Event-related desynchronization (ERD) in the alpha band during a hand mental rotation task. Neurosci. Lett. 541, 238–242. 10.1016/j.neulet.2013.02.036 23458675

[B9] Chen X. Bin G. Gao X. (2014). Effects of stimulus views on mental rotation of hands: an event-related potential study. Found. Pract. Appl. Cogn. Syst. Inform. Process. 215, 1–14. 10.1007/978-3-642-37835-5_1

[B10] Collins D. W. Kimura D. (1997). A large sex difference on a two-dimensional mental rotation task. Behav. Neurosci. 111, 845–849. 10.1037/0735-7044.111.4.845 9267662

[B11] Cooper L. A. Shepard R. N. (1973a). Chronometric studies of the rotation of mental images. Vis. Inform. Process. 75–176. 10.1016/B978-0-12-170150-5.50009-3

[B12] Cooper L. A. Shepard R. N. (1973b). The time required to prepare for a rotated stimulus. Mem. Cogn. 1, 246–250. 10.3758/BF03198104 24214553

[B13] Cummins T. R. Jiang C. Haddad G. G. (1993). Human neocortical excitability isdecreased during anoxia via sodium channel modulation. J. Clin. Invest. 91, 608–615. 10.1172/JCI116241 8381823 PMC287992

[B14] Dünnwald T. Kienast R. Niederseer D. Burtscher M. (2021). The use of pulse oximetry in the assessment of acclimatization to high altitude. Sensors 21:1263. 10.3390/s21041263 33578839 PMC7916608

[B15] Eva K. Hamm J. P. (2010). A model of rotated mirror/normal letter discriminations. Mem. Cogn. 38, 206–220. 10.3758/MC.38.2.206 20173193

[B16] Harris I. M. Miniussi C. (2003). Parietal lobe contribution to mental rotation demonstrated with rTMS. J. Cogn. Neurosci. 15, 315–323. 10.1162/089892903321593054 12729485

[B17] Heida T. Poppe N. R. de Vos C. C. van Putten M. J. A. M. van Vugt J. P. P. (2014). Event-related mu-rhythm desynchronization during movement observation is impaired in Parkinson's disease. Clin. Neurophysiol. 125, 1819–1825. 10.1016/j.clinph.2014.01.016 24560131

[B18] Heil M. (2002). The functional significance of ERP effects during mental rotation. Psychophysiology 39, 535–545. 10.1111/1469-8986.3950535 12236320

[B19] Heil M. Bajrić J. Rösler F. Hennighausen E. (1996). Event-related potentials during mental rotation: disentangling the contributions of character classification and image transformation. J. Psychophysiol. 10, 326–335.

[B20] Heil M. Rauch M. Hennighausen E. (1998). Response preparation begins before mental rotation is finished: evidence from event-related brain potentials. Acta. Psychol. 99, 217–232. 10.1016/S0001-6918(98)00012-2 9708033

[B21] Hochachka P. W. Clark C. M. Brown W. D. Stanley C. Stone C. K. Nickles R. J.. (1994). The brain at high altitude: hypometabolism as a defense against chronic hypoxia? J. Cereb. Blood Flow Metab. 14, 671–679. 10.1038/jcbfm.1994.84 8014215

[B22] Horst A. C. Jongsma M. L. Janssen L. K. Lier R. Steenbergen B. (2012). Different mental rotationstrategies reflected in the rotation related negativity. Psychophysiology 49, 566–573. 10.1111/j.1469-8986.2011.01322.x 22091978

[B23] Huster R. J. Geppert S. E. Lavallee C. F. Falkenstein M. Herrmann C. S. (2013). Electroencephalography of response inhibition tasks: functional networks and cognitive contributions. Int. J. Psychophysiol. 87, 217–233. 10.1016/j.ijpsycho.2012.08.001 22906815

[B24] Jagaroo V. (2004). Mental rotation and the parietal question in functional neuroimaging: a discussion of two views. Eur. J. Cogn. Psychol. 16, 717–728. 10.1080/09541440340000466

[B25] Jordan K. Heinze H. J. Lutz K. Kanowski M. Jäncke L. (2001). Cortical activations during the mental rotation of different visual objects. Neuroimage 13, 143–152. 10.1006/nimg.2000.0677 11133317

[B26] Klimesch W. (1999). EEG alpha and theta oscillations reflect cognitive and memory performance: a review and analysis. Brain Res. Rev. 29, 169–195. 10.1016/S0165-0173(98)00056-3 10209231

[B27] Klimesch W. Doppelmayr M. Hanslmayr S. (2006). Upper alpha ERD and absolute power: their meaning for memory performance. Prog. Brain Res. 159, 151–165. 10.1016/S0079-6123(06)59010-7 17071229

[B28] Klimesch W. Sauseng P. Hanslmayr S. (2007). EEG alpha oscillations: the inhibition-timing hypothesis. Brain Res. Rev. 53, 63–88. 10.1016/j.brainresrev.2006.06.003 16887192

[B29] Kolev V. Yordanova J. Schürmann M. Başar E. (1999). Event-related alpha oscillations in task processing. Clin. Neurophysiol. 110, 1784–1792. 10.1016/S1388-2457(99)00105-4 10574293

[B30] Lindeis A. E. Nathoo A. Fowler B. (1996). An AFM investigation of the effects of acutehypoxia on mental rotation. Ergonomics 39, 278–284. 10.1080/00140139608964458 8851532

[B31] Lyu Y. Guo X. Bekrater-Bodmann R. Flor H. Tong S. (2016). Phantom limb perception interferes with motor imagery after unilateral upper-limb amputation. Sci. Rep. 6:21100. 10.1038/srep21100 26879749 PMC4754632

[B32] Ma H. Li X. Liu M. Ma H. Zhang D. (2018). Mental rotation effect on adult immigrants with long-term exposure to high altitude in Tibet: an ERP study. Neuroscience 386, 339–350. 10.1016/j.neuroscience.2018.06.038 30049664

[B33] Ma H. Wang Y. Wu J. Luo P. Han B. (2015a). Long-term exposure to high altitude affects response inhibition in the conflict-monitoring stage. Sci. Rep. 5:13701. 10.1038/srep13701 26324166 PMC4555177

[B34] Ma H. Wang Y. Wu J. Wang B. Guo S. Luo P.. (2015b). Long-term exposure to high altitude affects conflict control in the conflict-resolving stage. PLoS ONE 10:e0145246. 10.1371/journal.pone.0145246 26671280 PMC4682854

[B35] Ma H. Zhang D. Li X. Ma H. Wang N. Wang Y. (2019). Long-term exposure to high altitude attenuates verbal and spatial working memory: evidence from an event-related potential study. Brain Behav. 9:e01256. 10.1002/brb3.1256 30891949 PMC6456776

[B36] Ma Q. Hu L. Li J. Hu Y. Xia L. Chen X.. (2016). Different effects of hypoxia on mental rotation of normal and mirrored letters: evidence from the rotation-related negativity. PLoS ONE 11:e0154479. 10.1371/journal.pone.0154479 27144444 PMC4856360

[B37] Marillier M. Arnal P. J. Le Roux Mallouf T. Rupp T. Millet G. Y. Verges S. (2017). Effects of high-altitude exposure on supraspinal fatigue and corticospinal excitability and inhibition. Eur. J. Appl. Physiol. 117, 1747–1761. 10.1007/s00421-017-3669-y 28647868

[B38] Milivojevic B. Hamm J. P. Corballis M. C. (2009). Functional neuroanatomy of mental rotation. J. Cogn. Neurosci. 21, 945–959. 10.1162/jocn.2009.21085 18702586

[B39] Milivojevic B. Johnson B. W. Hamm J. P. Corballis M. C. (2003). Non-identical neural mechanisms for two types of mental transformation: event-related potentials during mental rotation and mental paper folding. Neuropsychologia 41, 1345–1356. 10.1016/S0028-3932(03)00060-5 12757907

[B40] Muthukumaraswamy S. D. Johnson B. W. (2004). Primary motor cortex activation during action observation revealed by wavelet analysis of the EEG. Clin. Neurophysiol. 115, 1760–1766. 10.1016/j.clinph.2004.03.004 15261854

[B41] Nam C. S. Jeon Y. Kim Y.-J. Lee I. Park K. (2011). Movement imagery-related lateralization of event-related (de) synchronization (ERD/ERS): motor-imagery duration effects. Clin. Neurophysiol. 122, 567–577. 10.1016/j.clinph.2010.08.002 20800538

[B42] Naro A. Maggio M. G. Latella D. Rosa G. L. Calabrò R. S. (2021). Does embodied cognition allow a better management of neurological diseases? A review on the link between cognitive language processing and motor function. Appl. Neuropsychol. Adult 1–12. 10.1080/23279095.2021.1890595. [Epub ahead of print]. 33683162

[B43] Nation D. A. Bondi M. W. Gayles E. Delis D. C. (2017). Mechanisms of memory dysfunction during high altitude hypoxia training in military aircrew. J. Int. Neuropsychol. Soc. 23, 1–10. 10.1017/S1355617716000965 27923417 PMC5472446

[B44] Neubauer A. C. Fink A. (2009). Intelligence and neural efficiency. Neurosci. Biobehav. Rev. 33, 1004–1023. 10.1016/j.neubiorev.2009.04.001 19580915

[B45] Neubauer A. C. Fink A. Grabner R. H. (2006). Sensitivity of alpha band ERD to individual differences in cognition. Prog. Brain Res. 159, 167–178. 10.1016/S0079-6123(06)59011-9 17071230

[B46] Neubauer J. A. Sunderram J. (2004). Oxygen-sensing neurons in the central nervous system. J. Appl. Physiol. 96, 367–374. 10.1152/japplphysiol.00831.2003 14660498

[B47] Núñez-Peña M. I. Aznar-Casanova J. A. (2009). Mental rotation of mirrored letters: evidence from event-related brain potentials. Brain Cogn. 69, 180–187. 10.1016/j.bandc.2008.07.003 18713660

[B48] Ozga W. K. Zapała D. Wierzgała P. Augustynowicz P. Porzak R. Wójcik G. M. (2018). Acoustic neurofeedback increases beta ERD during mental rotation task. Appl. Psychophysiol. Biofeedback 44, 103–115. 10.1007/s10484-018-9426-0 30565198 PMC6505495

[B49] Pascual-Marqui R. D. Esslen M. Kochi K. Lehmann D. (2002). Functional imaging with low-resolution brain electromagnetic tomography (LORETA): a review. Method Find. Exp. Clin. 24(Suppl C), 91–95. 12575492

[B50] Pearson J. Kosslyn S. M. (2015). The heterogeneity of mental representation: ending the imagery debate. Proc. Natl. Acad. Sci. U.S.A. 112, 10089–10092. 10.1073/pnas.1504933112 26175024 PMC4547292

[B51] Peng W. Hu L. Zhang Z. Hu Y. (2012). Causality in the association between P300 and alpha event-related desynchronization. PLoS ONE 7:e34163. 10.1371/journal.pone.0034163 22511933 PMC3325251

[B52] Pfurtscheller G. (2001). Functional brain imaging based on ERD/ERS. Vis. Res. 41, 1257–1260. 10.1016/S0042-6989(00)00235-2 11322970

[B53] Pfurtscheller G. Aranibar A. (1977). Event-related cortical desynchronization detected by power measurements of scalp EEG. Electroenceph. Clin. Neurophysiol. 42, 817–826. 10.1016/0013-4694(77)90235-8 67933

[B54] Pfurtscheller G. Brunner C. Schlögl A. Lopes da Silva F. H. (2006). Mu rhythm(de)synchronizationand EEG single-trial classification of different motor imagery tasks. Neuroimage 31, 153–159. 10.1016/j.neuroimage.2005.12.003 16443377

[B55] Pfurtscheller G. Lopes da Silva F. H. (1999). Event-related EEG/MEG synchronization and desynchronization: basic principles. Clin. Neurophysiol. 110, 1842–1857. 10.1016/S1388-2457(99)00141-8 10576479

[B56] Pfurtscheller G. Scherer R. Müller-Putz G. R. Lopes da Silva F. H. (2008). Short-lived brainstate after cued motor imagery in naive subjects. Eur. J. Neurosci. 28, 1419–1426. 10.1111/j.1460-9568.2008.06441.x 18973568

[B57] Riečanský I. Katina S. (2010). Induced EEG alpha oscillations are related to mental rotation ability: the evidence for neural efficiency and serial processing. Neurosci. Lett. 482, 133–136. 10.1016/j.neulet.2010.07.017 20637833

[B58] Rostrup E. Larsson H. B. W. Born A. P. Knudsen G. M. Paulson O. B. (2005). Changes in BOLD and ADC weighted imaging in acute hypoxia during sea-level and altitude adapted states. NeuroImage 28, 947–955. 10.1016/j.neuroimage.2005.06.032 16095921

[B59] Ruggiero L. Yacyshy A. F. Nettleton J. McNeil C. J. (2018). UBC-nepal expedition: acclimatization to high-altitude increases spinal motoneurone excitability during fatigue in humans. J. Physiol. 596, 3327–3339. 10.1113/JP274872 29130497 PMC6068218

[B60] Save E. Poucet B. (2000). Hippocampal-parietal cortical interactions in spatial cognition. Hippocampus 10, 491–499. 10.1002/1098-1063(2000)10:4<491::AID-HIPO16>3.0.CO;2-0 10985289

[B61] Schürmann M. Başar E. (2001). Functional aspects of alpha oscillations in theEEG. Int. J. Psychophysiol. 39, 151–158. 10.1016/S0167-8760(00)00138-0 11163894

[B62] Schürmann M. Başar-Eroglu C. Başar E. (1997). A possible role of evoked alpha in primary sensory processing: common properties of cat intracranial recordings and human EEG and MEG. Int. J. Psychophysiol. 26, 149–170. 10.1016/S0167-8760(97)00762-9 9203001

[B63] Seurinck R. Vingerhoets G. de Lange F. P. Achten E. (2004). Does egocentric mental rotation elicit sex differences? NeuroImage 23, 1440–1449. 10.1016/j.neuroimage.2004.08.010 15589108

[B64] Shepard R. N. Cooper L. A. (1982). Mental Images and Their Transformations. Cambridge: MIT Press.

[B65] Shepard R. N. Metzler J. (1971). Mental rotation of three-dimensional objects. Science 171, 701–703. 10.1126/science.171.3972.701 5540314

[B66] Takemi M. Masakado Y. Liu M. Ushiba J. (2013). Event-related desynchronization reflects downregulation of intracortical inhibition in human primary motor cortex. J. Neurophysiol. 110, 1158–1166. 10.1152/jn.01092.2012 23761697

[B67] Takemi M. Masakado Y. Liu M. Ushiba J. (2015). Sensorimotor event-related desynchronization represents the excitability of human spinal motoneurons. Neuroscience 297, 58–67. 10.1016/j.neuroscience.2015.03.045 25839147

[B68] Tzagarakis C. Ince N. F. Leuthold A. C. Pellizzer G. (2010). Beta-band activity during motor planning reflects response uncertainty. J. Neurosci. 30, 11270–11277. 10.1523/JNEUROSCI.6026-09.2010 20739547 PMC6633326

[B69] Virués-Ortega J. Bucks R. Kirkham F. J. Baldeweg T. Baya-Botti A. Hogan A. M. (2011). Changing patterns of neuropsychological functioning in children living at high altitude above and below 4000 m: a report from the Bolivian Children Living at Altitude (BoCLA) study. Dev. Sci. 14, 1185–1193. 10.1111/j.1467-7687.2011.01064.x 21884333

[B70] Wang Y. Ma H. Fu S. Guo S. Yang X. Luo P.. (2014). Long-term exposure to high altitude affects voluntary spatial attention at early and late processing stages. Sci. Rep. 4, 1–8. 10.1038/srep04443

[B71] Wang Z. Guo X. Lyu Y. Chen H. Tong S. (2016). Spatiotemporal differences of brain activation between internal and external strategies in mental rotation: a behavioral and ERD/ERS study. Neurosci. Lett. 623, 1–6. 10.1016/j.neulet.2016.04.061 27132083

[B72] Wijers A. A. Otten L. J. Feenstra S. Mulder G. Mulder L. J. M. (1989). Brain potentials during selective attention, memory search, and mental rotation. Psychophysiology 26, 452–467. 10.1111/j.1469-8986.1989.tb01951.x 2798695

[B73] Williams J. D. Rippon G. Stone B. M. Annett J. (1995). Psychophysiological correlates of dynamic imagery. Br. J. Psychol. 86, 283–300. 10.1111/j.2044-8295.1995.tb02562.x 7795946

[B74] Wilson M. H. Newman S. Imray C. H. (2009). The cerebral effects of ascent to high altitudes. Lancet Neurol. 8, 175–191. 10.1016/S1474-4422(09)70014-6 19161909

[B75] Yan J. Guo X. Jin Z. Sun J. Shen L. Tong S. (2012). Cognitive alterations in motor imagery process after left hemispheric ischemic stroke. PLoS ONE 7:e42922. 10.1371/journal.pone.0042922 22912763 PMC3415407

[B76] Yu L. Wang X. Lyu Y. Ding L. Jia J. Tong S.. (2020). Electrophysiological evidences for the rotational uncertainty effect in the hand mental rotation: an ERP and ERS/ERD study. Neuroscience 432, 205–215. 10.1016/j.neuroscience.2020.02.040 32135235

[B77] Zacks J. (2008). Neuroimaging studies of mental rotation: a meta-analysis and review. J. Cogn. Neurosci. 20, 1–19. 10.1162/jocn.2008.20013 17919082

[B78] Zhang D. Zhang X. Ma H. Wang Y. Ma H. Liu M. (2018). Competition among the attentional networks due to resource reduction in tibetan indigenous residents: evidence from event-related potentials. Sci. Rep. 8:610. 10.1038/s41598-017-18886-7 29330442 PMC5766594

[B79] Zhang J. Zhang H. Li J. Chen J. Han Q. Lin J.. (2013). Adaptive modulation of adult brain gray and white matter to high altitude: structural MRI studies. PLoS ONE 8:e68621. 10.1371/journal.pone.0068621 23874692 PMC3712920

